# Association of 25-hydroxyvitamin D with all-cause and cardiovascular mortality among individuals with sarcopenia: evidence from the NHANES 2001–2006

**DOI:** 10.3389/fnut.2025.1562897

**Published:** 2025-05-16

**Authors:** Chen Fu, Zhongxin Zhu, Yijie Mao, Wenjuan Wei

**Affiliations:** ^1^Department of Cardiology, The First People's Hospital of Xiaoshan District, Xiaoshan Affiliated Hospital of Wenzhou Medical University, Hangzhou, China; ^2^Department of Scientific Research, The First People's Hospital of Xiaoshan District, Xiaoshan Affiliated Hospital of Wenzhou Medical University, Hangzhou, China; ^3^Department of Training, Hangzhou Xiaoshan NO.4 Secondary Vocational School, Hangzhou, China

**Keywords:** 25(OH)D, sarcopenia, mortality, cardiovascular risk, NHANES

## Abstract

**Background:**

Sarcopenia, defined as the progressive decline in muscular mass and physical power, poses significant health risks, particularly among aging populations.

**Methods:**

Utilizing data from the National Health and Nutrition Examination Survey (NHANES) spanning 2001–2006, we employed multivariable Cox proportional hazards models to evaluate the relationship between serum 25-hydroxyvitamin D [25(OH)D] concentration and mortality outcomes, adjusting for multiple covariates. We additionally performed restricted cubic spline and threshold analyses using both linear and non-linear regression models to assess dose–response relationships and to explore the continuous effects of 25(OH)D on mortality outcomes. Stratified and sensitivity analyses were conducted to strengthen the reliability of our findings.

**Results:**

A total of 1,666 participants diagnosed with sarcopenia were included in the analysis. Our results indicated a significant non-linear association between 25(OH)D concentration and both all-cause mortality and cardiovascular (CVD) mortality. Notably, threshold analyses revealed inflection points at 62.563 nmol/L for all-cause mortality and 47.367 nmol/L for CVD mortality, suggesting a plateau in protective effects at higher vitamin D levels. Both stratified and sensitivity analyses revealed no significant interactions across different subgroups.

**Conclusion:**

These findings emphasize the importance of maintaining adequate serum 25(OH)D concentration to mitigate mortality risk among sarcopenic individuals. Further research is needed to clarify the underlying mechanisms and to establish optimal vitamin D concentration for health benefits.

## 1 Introduction

Sarcopenia, defined as the progressive decline in muscular mass and physical power, has risen to prominence as a major public health challenge, particularly among aging populations. The European Working Group on Sarcopenia in Older People (EWGSOP) identified sarcopenia as a syndrome associated with increased morbidity, functional decline, and mortality (1). The prevalence of sarcopenia is estimated to affect approximately 10–16% of older adults, with rates increasing with age and the presence of comorbidities (2). As the global population ages, understanding the factors contributing to sarcopenia and its associated health-related risks becomes increasingly critical. Low levels of 25-hydroxyvitamin D [25(OH)D], the primary circulating form of vitamin D, have been correlated with an elevated risk of sarcopenia and associated adverse outcomes (3, 4).

Serum 25(OH)D is a critical biomarker for evaluating vitamin D status in the human body. This metabolite is synthesized in the liver through the hydroxylation of vitamin D, which can be obtained either from dermal synthesis upon exposure to ultraviolet B radiation or from dietary sources. Following its synthesis, serum 25(OH)D is further converted into its biologically active form, calcitriol, in the kidneys. This metabolite is essential for regulating calcium and phosphorus homeostasis, as well as influencing a wide range of biological processes, including immune system function and muscle health. Recent research has highlighted the potential association between optimal concentration of 25(OH)D and a decrease in the risk of various chronic diseases, including cardiovascular diseases (CVD), autoimmune disorders, and certain cancers, as well as improvements in overall mortality outcomes (5–11). Despite the growing body of literature linking vitamin D to health outcomes, there are still uncertainties regarding the specific mechanisms through which vitamin D influences mortality, particularly in the context of sarcopenia. Some studies suggest a non-linear relationship between 25(OH)D concentration and health outcomes, indicating that both deficiency and excess may be detrimental (12, 13). Identifying critical thresholds of 25(OH)D that correlate with improved health outcomes is essential for developing effective public health strategies and clinical guidelines.

The National Health and Nutrition Examination Survey (NHANES) is a cross-sectional survey that collects comprehensive health and nutritional data from a representative sample of the U.S. population, allowing for robust epidemiological analyses. Previous studies utilizing NHANES data have demonstrated significant associations between low 25(OH)D levels and various health outcomes, including CVD and all-cause mortality (14–16). However, the specific relationship between 25(OH)D levels and mortality among individuals with sarcopenia remains underexplored.

This study aims to examine the association between serum 25(OH)D concentration and mortality outcomes in individuals with sarcopenia, using data from the NHANES collected between 2001 and 2006. By investigating this relationship, we seek to contribute to the existing literature on the role of vitamin D in mortality risk among individuals with sarcopenia, thereby informing future research directions and potential interventions.

## 2 Methods

### 2.1 Study population

**Figure 1:**
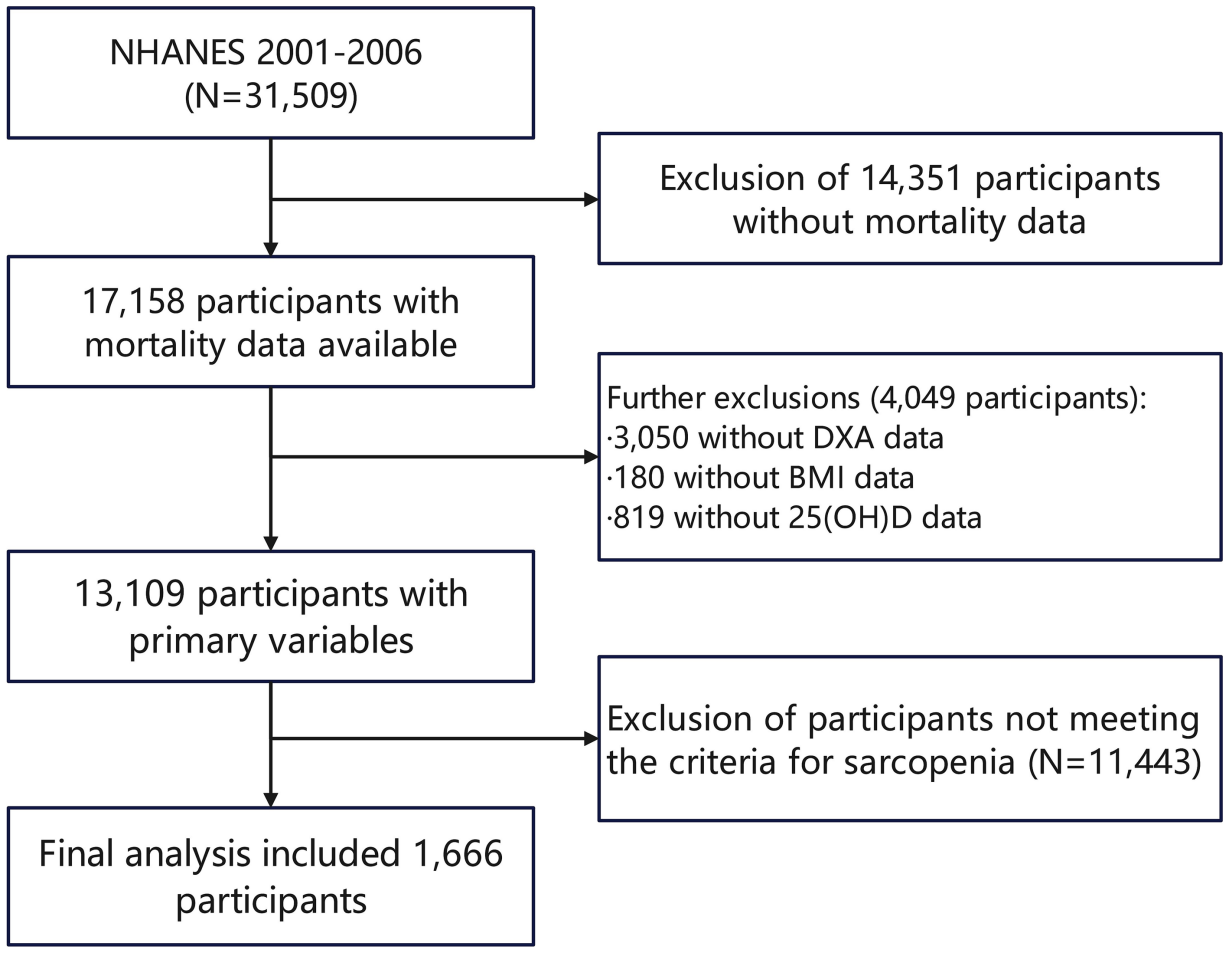
Flowchart diagram of participants’ selection in this study.

The study population for this analysis comprises participants from the NHANES datasets spanning the years 2001–2006, totaling 31,509 individuals. The ethics review board of the National Center for Health Statistics approved all NHANES protocols. All participants provided informed consent and adhered to the established study protocols, ensuring ethical compliance and the reliability of the data collected. During the initial screening process, 17,158 participants with available mortality data were identified, with 14,351 excluded specifically due to missing mortality data. Further screening of the study population based on the primary variables of interest resulted in the removal of 3,050 participants without DXA data, 180 without body mass index (BMI) data, and 819 lacking 25(OH)D data. After these exclusions, 13,109 participants remained eligible for analysis. Ultimately, after assessing sarcopenia using threshold values of the sarcopenia index (for the values of the threshold, refer to Methods, Section 2.2), the final analytic cohort included 1,666 participants who fulfilled all the eligibility criteria. (Figure 1).

### 2.2 Definition of sarcopenia

Sarcopenia diagnosis was based on precise measurements obtained through DXA (17), a robust technique recognized for assessing body composition. The primary metrics evaluated included the total appendicular skeletal muscle mass, which quantifies the muscles of the limbs, and specifically, the appendicular lean mass (ALM). The assessment process commenced with the calculation of ALM, derived from DXA scans that provided detailed insights into the muscle quality of each participant. To contextualize this data, the sarcopenia index was calculated using a formula that divides the ALM in kilograms by the BMI expressed in kg/m^2^. This methodological approach enabled a standardized evaluation of muscle mass relative to the individual’s body composition. Sarcopenia was ultimately defined using gender-specific thresholds for the sarcopenia index: the critical cut-off values are established at 0.512 for women and 0.789 for men (18). Participants who failed to reach these criteria were designated as sarcopenic and subsequently selected as the study population.

### 2.3 Serum 25(OH)D measurement

The measurement of 25(OH)D (25(OH)D) from 2001 to 2006 underwent specific methodological transitions and standardization. Initially, serum 25(OH)D concentration were determined using radioimmunoassay (RIA). These RIA data were then converted through a series of complex statistical analyses and experimental validations, making them equivalent to those measured by liquid chromatography–tandem mass spectrometry (LC–MS/MS) (19). Based on the Clinical Practice Guidelines published by the Endocrine Society (20), serum 25(OH)D concentration were stratified into four distinct categories: severe deficiency (<25.00 nmol/L), deficiency (25.00–49.99 nmol/L), insufficiency (50.00–74.99 nmol/L), and sufficiency (≥75.00 nmol/L).

### 2.4 Assessment of mortality and follow-up

Mortality outcomes were assessed through a rigorous follow-up process, which involved linking NHANES data with the National Death Index (NDI). This linkage allowed for the accurate determination of vital status and cause of death for participants over a follow-up period that extended to December 31, 2019. The follow-up was conducted annually, ensuring that any changes in mortality status were promptly recorded. The assessment of mortality included both all-cause mortality and cause-specific mortality, particularly focusing on CVD mortality. Causes of death were classified according to the International Classification of Diseases (ICD) codes, allowing for standardized reporting and analysis. In particular, CVD mortality were coded using the ICD-10 ranges I00-I09, I11, I13, and I20-I51 (21).

### 2.5 Covariates definition

To ensure the robustness of our analysis on the association between 25(OH)D and risk of mortality, we carefully selected a series of variables that might have confounding effects on the association: Demographic Characteristics, which encompassed age distribution, gender, and racial diversity. Socioeconomic Status including educational attainment, categorized into three levels (below high school, high school, and above high school), and poverty income ratio (PIR), categorized into quartiles (≤1.35, 1.35–1.85, and >1.85). Body measurement comprised BMI, which was categorized as ≤24.9 kg/m^2^, 25.0–29.9 kg/m^2^ and ≥30.0 kg/m^2^. Chronic diseases were evaluated through self-reported medical histories and laboratory assessments, focusing on key conditions such as hypertension, diabetes, coronary heart disease (CHD), stroke, bronchitis and cancer. Behavioral Factors included smoking history (consuming at least 100 cigarettes in their lifetime or not), and drinking status, categorized into four groups: none, mild–moderate (up to one drink per day for women and up to two for men), heavy (more than mild–moderate consumption), and former drinkers (22). The analysis encompassed several laboratory biomarkers: glycohemoglobin, hemoglobin, serum albumin, alanine aminotransferase (ALT), aspartate aminotransferase (AST), estimated glomerular filtration rate (eGFR), high-density lipoprotein cholesterol (HDL-C) and total cholesterol (TC). For detailed procedures and criteria for evaluating covariates, refer to the NHANES data collection protocols (23).

### 2.6 Statistical analysis

Statistical analyses were conducted using R software (version 4.3.2) and EmpowerStats (version 4.2). Descriptive statistics were computed for baseline characteristics, stratified by serum 25(OH)D levels. Continuous data are presented as means ± standard deviations (SD), while categorical data are expressed as percentages. To evaluate the association between serum 25(OH)D concentration and mortality outcomes, multivariable Cox proportional hazards models were employed. Three distinct models were developed to systematically assess the impact of different covariates on mortality risk: Model 1 adjusted for demographic factors, including gender, age, race, education level, and PIR. Model 2 further adjusted for BMI, smoking history, drinking status and chronic diseases such as hypertension, diabetes, CHD, stroke, bronchitis and cancer. Model 3 included further adjustments for laboratory biomarkers including glycohemoglobin, hemoglobin, serum albumin, ALT, AST, eGFR, TC and HDL-C. Hazard ratios (HR) and 95% confidence intervals (CI) were computed from these models, with statistical significance determined at *p* < 0.05. Non-linear relationships between serum 25(OH)D concentration and mortality risk were assessed utilizing restricted cubic splines and piecewise Cox regression models, with inflection points identified through the log-likelihood ratio test. Stratified analyses were performed to evaluate the association between serum 25(OH)D concentration and mortality across various demographic and health-related groups. These subgroups were defined based on gender, race, age distribution, PIR ranges, BMI classification, smoking and drinking status, and the presence or absence of hypertension, diabetes, CHD, and stroke. Sensitivity analyses were conducted by excluding participants with a history of CHD, stroke, bronchitis and cancer. Additionally, individuals who died within 2 years were excluded, along with participants who had extreme values of 25(OH)D (mean ± 3 SD). Missing values in covariates were addressed using multiple imputation with 10 iterations. The imputation model included all study variables to preserve multivariate relationships.

## 3 Results

### 3.1 Baseline characteristics

Table 1 summarizes the baseline characteristics of the participants. This study enrolled 1,666 participants with a mean age of 59.10 ± 18.34 years; 52.94% of whom were males. Among these participants, 44.96% had vitamin D deficiency (< 50.00 nmol/L), and 86.68% had vitamin D insufficiency (< 75.00 nmol/L). Significant differences were observed in demographic profiles and baseline clinical characteristics between participants with varying 25(OH)D concentrations. Specifically, individuals with higher vitamin D concentration exhibited a significantly greater proportion of males, elderly individuals, non-Hispanic White people, and those with higher educational attainment and a lower BMI. Similarly, the proportion of individuals diagnosed with CHD and cancer was significantly higher in the higher 25(OH)D group compared to the lower 25(OH)D group.

**Table 1:** Baseline characteristics of selected individuals according to 25(OH)D categories.

Serum 25(OH)D categorical (nmol/L)	Total	<25.00	25.00–49.99	50.00–74.99	≥75.00	*p*-value
*N* (%)	1,666 (100%)	78 (4.68%)	671 (40.28%)	695 (41.72%)	222 (13.33%)	
Age (years)	59.10 ± 18.34	58.01 ± 18.73	55.39 ± 18.78	61.27 ± 17.80	63.87 ± 16.36	<0.001
Gender (%)						0.001
Male	882 (52.94%)	27 (34.62%)	340 (50.67%)	386 (55.54%)	129 (58.11%)	
Female	784 (47.06%)	51 (65.38%)	331 (49.33%)	309 (44.46%)	93 (41.89%)	
Race (%)						<0.001
Mexican American	685 (41.12%)	38 (48.72%)	343 (51.12%)	254 (36.55%)	50 (22.52%)	
Other Hispanic	84 (5.04%)	3 (3.85%)	40 (5.96%)	32 (4.60%)	9 (4.05%)	
Non-Hispanic White	760 (45.62%)	16 (20.51%)	219 (32.64%)	368 (52.95%)	157 (70.72%)	
Non-Hispanic Black	79 (4.74%)	16 (20.51%)	42 (6.26%)	18 (2.59%)	3 (1.35%)	
Other race or multi- racial	58 (3.48%)	5 (6.41%)	27 (4.02%)	23 (3.31%)	3 (1.35%)	
Educational degree (%)						0.010
Below high school	761 (45.68%)	34 (43.59%)	329 (49.03%)	319 (45.90%)	79 (35.59%)	
High school	389 (23.35%)	21 (26.92%)	151 (22.50%)	148 (21.29%)	69 (31.08%)	
Above high school	516 (30.97%)	23 (29.49%)	191 (28.46%)	228 (32.81%)	74 (33.33%)	
PIR (%)						0.384
≤1.35	647 (38.84%)	32 (41.03%)	270 (40.24%)	272 (39.14%)	73 (32.88%)	
>1.35, ≤ 1.85	218 (13.09%)	6 (7.69%)	85 (12.67%)	96 (13.81%)	31 (13.96%)	
>1.85	801 (48.08%)	40 (51.28%)	316 (47.09%)	327 (47.05%)	118 (53.15%)	
BMI (kg/m^2^)	31.68 ± 6.71	35.41 ± 9.15	32.26 ± 7.08	31.19 ± 6.31	30.10 ± 4.92	<0.001
Cigarettes consumption>100 (%)						0.064
Yes	820 (49.22%)	37 (47.44%)	305 (45.45%)	359 (51.65%)	119 (53.60%)	
No	846 (50.78%)	41 (52.56%)	366 (54.55%)	336 (48.35%)	103 (46.40%)	
Drinking status						0.004
Never	307 (18.43%)	15 (19.23%)	142 (21.16%)	111 (15.97%)	39 (17.57%)	
Mild-to-moderate	467 (28.03%)	14 (17.95%)	166 (24.74%)	213 (30.65%)	74 (33.33%)	
Heavy	412 (24.73%)	24 (30.77%)	185 (27.57%)	152 (21.87%)	51 (22.97%)	
Former	480 (28.81%)	25 (32.05%)	178 (26.53%)	219 (31.51%)	58 (26.13%)	
History of diseases						
Hypertension (%)						0.004
Yes	751 (45.08%)	41 (52.56%)	269 (40.09%)	326 (46.91%)	115 (51.80%)	
No	915 (54.92%)	37 (47.44%)	402 (59.91%)	369 (53.09%)	107 (48.20%)	
Coronary heart disease (%)						0.002
Yes	139 (8.34%)	9 (11.54%)	36 (5.37%)	66 (9.50%)	28 (12.61%)	
No	1,527 (91.66%)	69 (88.46%)	635 (94.63%)	629 (90.50%)	194 (87.39%)	
Diabetes mellitus						0.080
Yes	357 (21.43%)	23 (29.49%)	145 (21.61%)	153 (22.01%)	36 (16.22%)	
No	1,309 (78.57%)	55 (70.51%)	526 (78.39%)	542 (77.99%)	186 (83.78%)	
Stroke (%)						0.003
Yes	100 (6.00%)	10 (12.82%)	31 (4.62%)	38 (5.47%)	21 (9.46%)	
No	1,566 (94.00%)	68 (87.18%)	640 (95.38%)	657 (94.53%)	201 (90.54%)	
Bronchitis						0.055
Yes	141 (8.46%)	11 (14.10%)	44 (6.56%)	67 (9.64%)	19 (8.56%)	
No	1,525 (91.54%)	67 (85.90%)	627 (93.44%)	628 (90.36%)	203 (91.44%)	
Cancer						0.002
Yes	167 (10.02%)	5 (6.41%)	48 (7.15%)	82 (11.80%)	32 (14.41%)	
No	1,499 (89.98%)	73 (93.59%)	623 (92.85%)	613 (88.20%)	190 (85.59%)	
Laboratory features						
Glycohemoglobin	5.97 ± 1.29	6.19 ± 1.42	6.02 ± 1.44	5.94 ± 1.16	5.85 ± 1.09	0.146
Hemoglobin	14.37 ± 1.50	13.68 ± 1.73	14.28 ± 1.55	14.49 ± 1.43	14.48 ± 1.43	<0.001
Albumin	41.41 ± 3.16	39.71 ± 3.59	41.39 ± 3.15	41.57 ± 3.11	41.56 ± 3.07	<0.001
ALT	27.05 ± 19.04	26.19 ± 18.53	27.96 ± 22.60	26.69 ± 16.64	25.72 ± 13.70	0.228
AST	25.92 ± 12.30	26.83 ± 15.80	25.89 ± 13.59	25.97 ± 11.48	25.54 ± 8.85	0.026
eGFR	65.98 ± 27.18	65.01 ± 26.88	71.15 ± 29.23	63.65 ± 25.44	57.98 ± 23.03	<0.001
Total cholesterol (mg/ dL)	50.56 ± 14.62	51.41 ± 16.40	49.07 ± 13.85	51.19 ± 14.68	52.79 ± 15.62	0.003
HDL- cholesterol (mg/ dL)	204.70 ± 44.92	200.63 ± 51.46	204.05 ± 47.29	204.83 ± 42.51	207.70 ± 42.55	0.267
All-cause death	695 (41.72%)	42 (53.85%)	247 (36.81%)	297 (42.73%)	109 (49.10%)	<0.001
CVD death	182 (10.92%)	15 (19.23%)	60 (8.94%)	79 (11.37%)	28 (12.61%)	0.029

Mean ± SD for continuous variables: the *p*-value was calculated by a linear regression model. % for categorical variables; the *p*-value was calculated by a chi-square test. 25(OH)D, 25-hydroxyvitamin D; PIR, poverty income ratio; BMI, body mass index; ALT, alanine aminotransferase; AST, aspartate aminotransferase; eGFR, estimated glomerular filtration rate; HDL, high density lipoprotein; CVD, cardiovascular disease.

### 3.2 Association between 25(OH)D and risk of mortality

During an average follow-up period of 151.9 months, there were 695 all-cause deaths, including 182 deaths related to CVD. Table 2 elucidates the association between serum 25(OH)D concentrations and mortality outcomes in participants with sarcopenia, using a multivariable Cox regression analysis with three models. In model 1, a significant inverse association was observed between 25(OH)D concentration and all-cause mortality. Specifically, for each one-unit increase in ln-transformed 25(OH)D, the HR was 0.57 (95% CI: 0.46, 0.70; *p* < 0.0001). After further adjustments in Model 3, the HR for all-cause mortality changed to 0.63 (95% CI: 0.51, 0.78; *p* < 0.0001) for each one-unit increase in ln-transformed 25(OH)D. The categorical HRs were 0.59 (95% CI: 0.41, 0.83; *p* = 0.0027) for concentration ≥25 and <50 nmol/L, 0.46 (95% CI: 0.32, 0.66; *p* < 0.0001) for concentration ≥50 and <75 nmol/L, and 0.52 (95% CI: 0.36, 0.77; *p* = 0.0011) for concentration ≥75 nmol/L. Regarding CVD mortality, Model 1 indicated an HR of 0.49 (95% CI: 0.32, 0.75; *p* = 0.0150) for each one-unit increase in ln-transformed 25(OH)D. Similarly, Model 3 reported an HR of 0.62 (95% CI: 0.41, 0.95; *p* = 0.0266) for each one-unit increase in ln-transformed 25(OH)D, with HRs of 0.33 (95% CI: 0.18, 0.62; *p* = 0.0005) for concentration ≥25 to <50 nmol/L, 0.27 (95% CI: 0.15, 0.51; *p* < 0.0001) for concentration ≥50 to <75 nmol/L, and 0.31 (95% CI: 0.15, 0.62; *p* = 0.0010) for concentration ≥75 nmol/L.

**Table 2:** Multivariable Cox regression analysis for mortality among participants with sarcopenia.

	Serum 25(OH)D concentration (nmol/L)					Per one-unit increment in ln-transformed 25(OH)D
Model	<25.00	25.00–49.99	50.00–74.99	≥75.00	*p* for trend	
All-cause mortality						
Number of death (%)	42 (53.85%)	247 (36.81%)	297 (42.73%)	109 (49.10%)		
Model 1	Reference	0.46 (0.33, 0.65) < 0.0001	0.36 (0.25, 0.51) < 0.0001	0.41 (0.28, 0.60) < 0.0001	<0.0001	0.57 (0.46, 0.70) < 0.0001
Model 2	Reference	0.52 (0.37, 0.75) 0.0003	0.41 (0.28, 0.58) < 0.0001	0.48 (0.33, 0.71) 0.0002	0.0007	0.61 (0.49, 0.75) < 0.0001
Model 3	Reference	0.59 (0.41, 0.83) 0.0027	0.46 (0.32, 0.66) < 0.0001	0.52 (0.36, 0.77) 0.0011	0.0010	0.63 (0.51, 0.78) < 0.0001
CVD mortality						
Number of death (%)	15 (19.23%)	60 (8.94%)	79 (11.37%)	28 (12.61%)		
Model 1	Reference	0.24 (0.13, 0.43) < 0.0001	0.18 (0.10, 0.33) < 0.0001	0.20 (0.10, 0.40) < 0.0001	0.0149	0.49 (0.32, 0.75) 0.0009
Model 2	Reference	0.28 (0.15, 0.51) < 0.0001	0.21 (0.11, 0.40) < 0.0001	0.25 (0.13, 0.50) < 0.0001	0.0789	0.56 (0.37, 0.86) 0.0084
Model 3	Reference	0.33 (0.18, 0.62) 0.0005	0.27 (0.15, 0.51) < 0.0001	0.31 (0.15, 0.62) 0.0009	0.1413	0.62 (0.41, 0.95) 0.0266

Model 1: adjust for gender, age, race, education, PIR. Model 2: adjust for gender, age, race, education, PIR, BMI, smoking history, drinking status, hypertension, CHD, diabetes, stroke, bronchitis and cancer. Model 3: adjust for gender, age, race, education, PIR, BMI, smoking history, drinking status, hypertension, CHD, diabetes, stroke, bronchitis, cancer, serum albumin, ALT, AST, eGFR, glycohemoglobin, hemoglobin, TC and HDL-C. 95% CI, 95% confidence interval; OR, odds ratio; 25(OH)D, 25-hydroxyvitamin D; CVD, cardiovascular disease. *p* < 0.05 was considered statistically significant.

### 3.3 Non-linear relationship and threshold analysis

**Figure 2:**
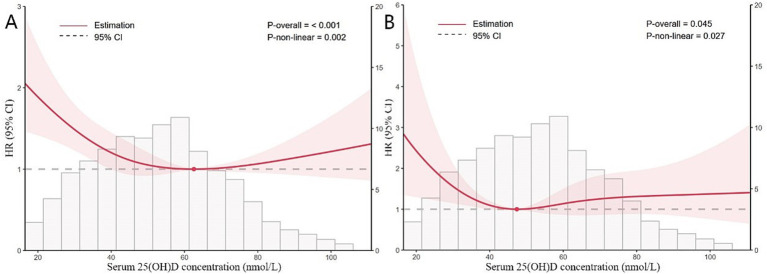
Restricted cubic splines of nonlinear relationships between 25(OH)D and all-cause **(A)** and CVD **(B)** mortality in participants with sarcopenia. The analysis was adjusted for gender, age, race, education, PIR, BMI, smoking history, drinking status, hypertension, CHD, diabetes, stroke, bronchitis, cancer, serum albumin, ALT, AST, eGFR, glycohemoglobin, hemoglobin, TC and HDL-C.

Data from the NHANES cohort were analyzed using a two-piecewise Cox proportional hazards model alongside restricted cubic splines. Inflection points were identified at 62.563 nmol/L for all-cause mortality and 47.367 nmol/L for CVD mortality, marking significant thresholds where the risk profile changes (Table 3). In K-segment effect 1, which encompasses lower levels of 25(OH)D, a significant protective association is observed. The HRs are 0.98 (95% CI: 0.98, 0.99; *p* < 0.0001) for all-cause mortality and 0.97 (95% CI: 0.94, 0.99; *p* = 0.0016) for CVD mortality. However, the transition to K-segment effect 2 marks a critical inflection point in the analysis. In this segment, the HRs slightly increase to 1.01 (95% CI: 1.00, 1.01; *p* = 0.1888) for all-cause mortality and 1.01 (95% CI: 0.99, 1.02; *p* = 0.3362) for CVD mortality. This shift indicates a potential plateau in the protective effect of 25(OH)D, suggesting that higher levels may not confer additional benefits and could even pose risks (Figure 2).

**Table 3:** Threshold effect for the association between 25(OH)D and mortality.

Threshold effect	All-cause mortality	CVD mortality
Infection points (K)	62.563	47.367
K-segment effect 1	0.98 (0.98, 0.99) < 0.0001	0.97 (0.94, 0.99) 0.0016
>K-segment effect 2	1.01 (1.00, 1.01) 0.1888	1.01 (0.99, 1.02) 0.3362
Effect size difference of 2 versus 1	1.02 (1.01, 1.03) 0.0009	1.04 (1.01, 1.07) 0.0058
Log likelihood ratio tests	0.001	0.007

HRs were calculated based on multivariable Cox proportional hazards regression model adjust for gender, age, race, education, PIR, BMI, smoking history, drinking status, hypertension, CHD, diabetes, stroke, bronchitis, cancer, serum albumin, ALT, AST, eGFR, glycohemoglobin, hemoglobin, TC and HDL-C.

### 3.4 Stratified analysis and sensitivity analysis

**Figure 3:**
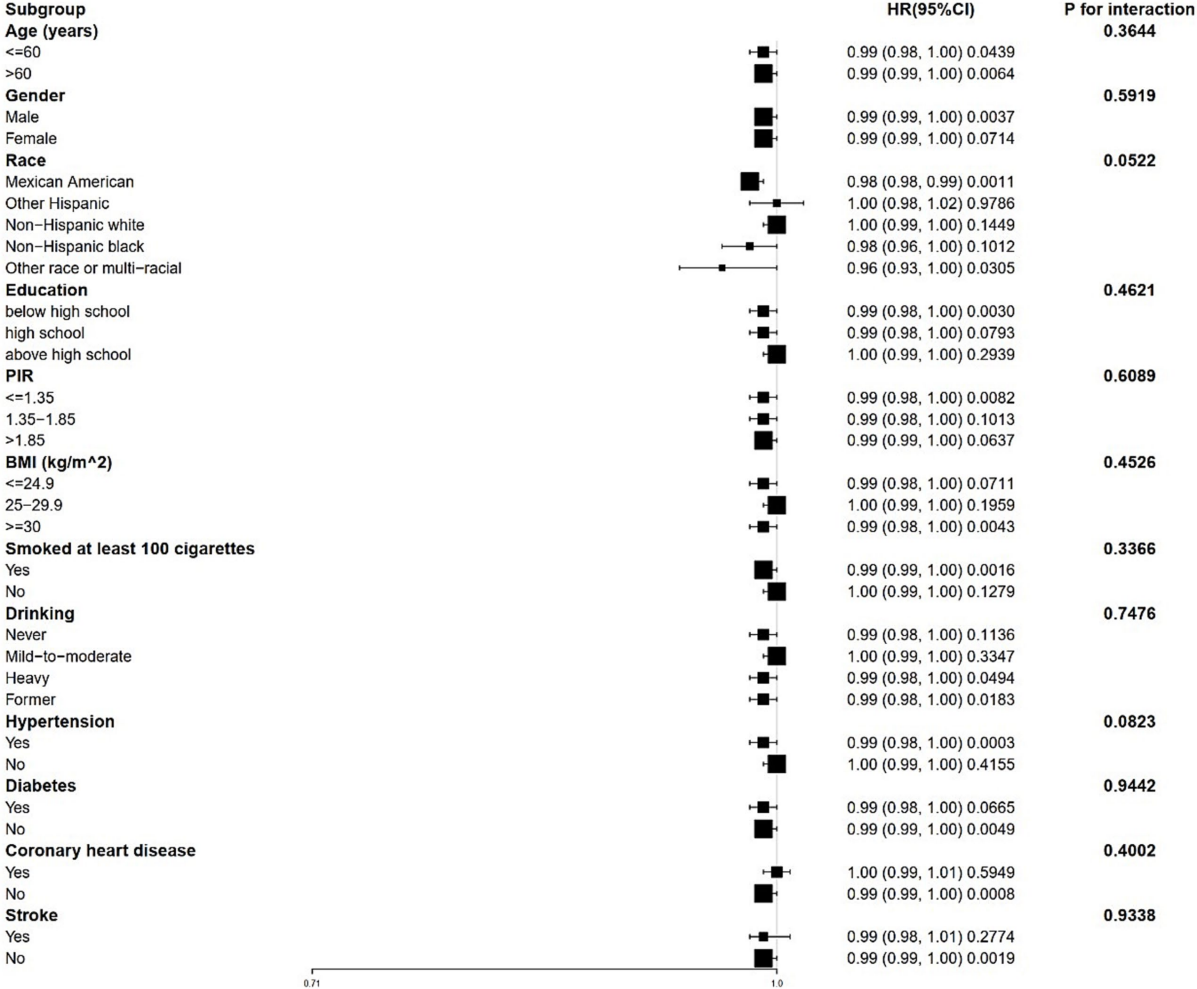
Forest plot for stratified analysis for the association between 25(OH)D and all-cause mortality. PIR, poverty income ratio; BMI, body mass index.

**Figure 4:**
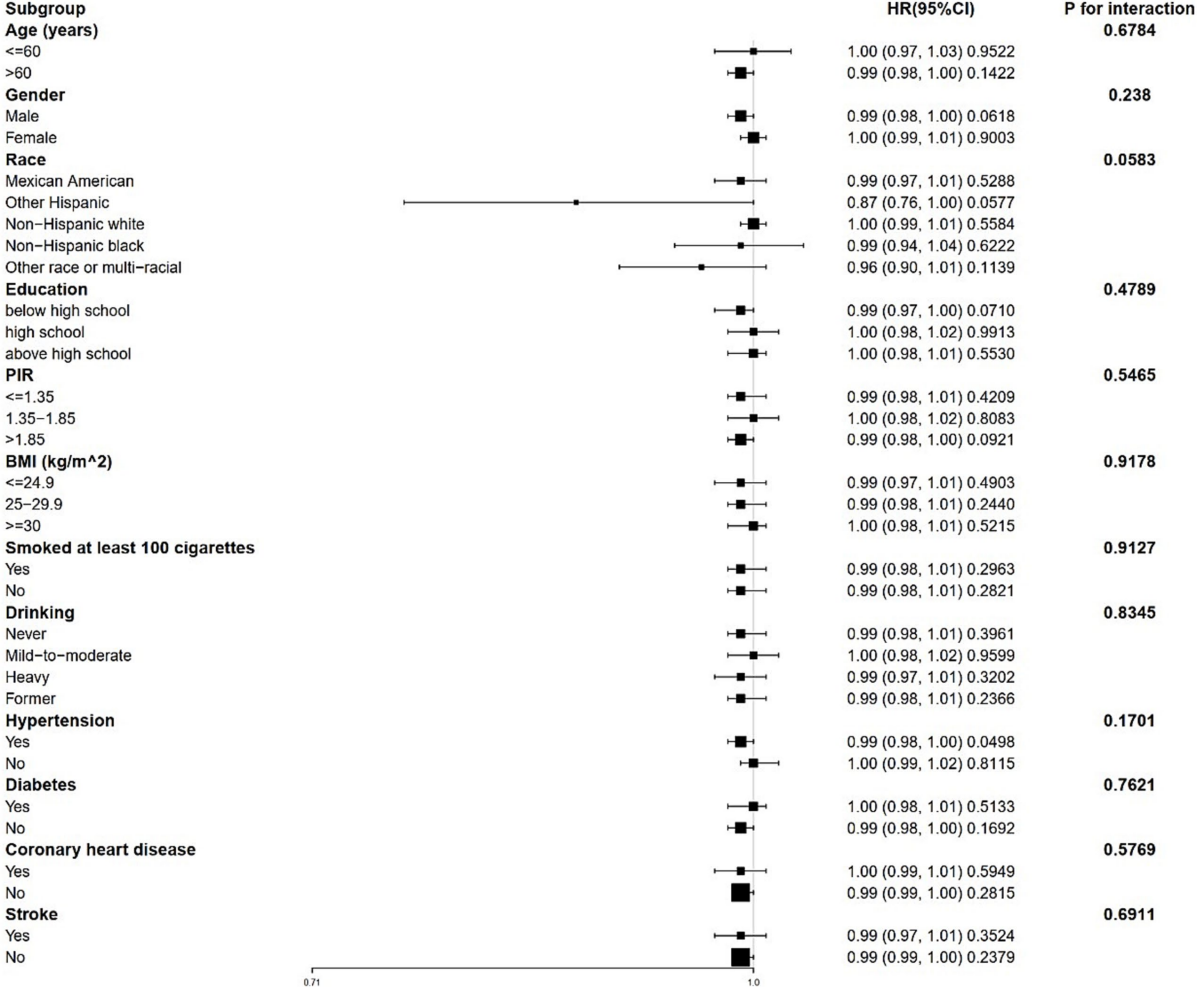
Forest plot for stratified analysis for the association between 25(OH)D and CVD mortality. PIR, poverty income ratio; BMI, body mass index.

Stratified analyses revealed that the correlation between 25(OH)D and both all-cause and CVD mortality remains consistent across different subgroups (Figures 3, 4). This indicates that variables such as gender, race, age, educational levels, PIR categories, BMI categories, hypertension, diabetes, CHD, stroke, smoking history and drinking status did not exert a significant influence on this positive correlation (*p* > 0.05 for all subgroups). Among these variables, race presented *p*-values that were close to the significance threshold. Specifically, Mexican Americans exhibited a HR of 0.98 (95% CI: 0.98–0.99, *p* = 0.0011) for all-cause mortality, while Other Hispanic group demonstrated a lower HR of 0.87 (95% CI: 0.76–1.00, *p* = 0.0577) for CVD mortality.

Sensitivity analyses were performed following the exclusion of participants with a history of CHD, stroke, bronchitis and cancer. No significant differences were observed compared to the original results (Supplementary Table S1–S4). Similarly, when individuals who died within 2 years were excluded from the analysis, the relationship between 25(OH)D concentration and mortality remained unchanged (Supplementary Table S5). After excluding extreme values (mean ± 3 SD) of 25(OH)D, the result also remain unchanged (Supplementary Table S6). Additionally, Supplementary Table S7 provides the proportion of missing data, while Supplementary Figures 1, 2 demonstrate the consistency of the results with multiple imputations.

## 4 Discussion

The present study provides compelling evidence of the association between serum 25(OH)D concentration and mortality outcomes in a cohort of participants from NHANES. Our findings indicate that lower concentration of 25(OH)D are significantly associated with increased risks of all-cause and CVD mortality among individuals diagnosed with sarcopenia. The findings reveal that for each one-unit increase in ln-transformed 25(OH)D, the HR for all-cause mortality is 0.63 and for CVD mortality is 0.62, indicating a protective effect of higher vitamin D concentration. This association remains robust even after adjusting for various covariates. Notably, the identification of inflection points at 62.563 nmol/L for all-cause mortality and 47.367 nmol/L for CVD mortality suggests a potential threshold effect, beyond which the benefits of increased vitamin D levels plateau. This finding aligns with previous research indicating that while low vitamin D concentration are detrimental, excessively high concentration may not confer additional protective benefits and could potentially pose risks (24).

Our results corroborate findings from several studies that have linked low serum 25(OH)D concentration to an increased mortality risk. For instance, a meta-analysis reported that individuals with low 25(OH)D concentration had a considerably greater risk of all-cause mortality compared to those with adequate concentration (13). This meta-analysis included data from various cohorts and highlighted the consistency of the association across different populations. Moreover, a systematic review by Autier et al. further supports our findings, indicating that low serum 25(OH)D concentration are associated with increased mortality risk in older adults (25). This review emphasized the importance of vitamin D in maintaining overall health and longevity, particularly in aging populations. Additionally, a recent study by Wan et al. demonstrated that higher 25(OH)D concentration were linked to lower mortality rates in a cohort of adults with diabetes, suggesting that monitoring and maintaining of 25(OH)D levels in diabetic patients is a potential intervention to reduce mortality risk (17). However, discrepancies exist in the literature regarding the optimal concentration of 25(OH)D for health benefits. While some studies propose that serum 25(OH)D concentrations above 75 nmol/L may be required for optimal health outcomes (19, 26–28), emerging guidelines recommend higher supplementation doses in elderly populations to achieve target levels of 75–125 nmol/L (29). Furthermore, higher doses of vitamin D supplementation, such as 50,000 IU per week or 100,000 IU per month of cholecalciferol, are not associated with additional toxicity (30). This contrasts with perspectives from the ‘Controversies in Vitamin D’ conference, which suggested that concentrations exceeding 50 nmol/L might suffice for general populations (31). Our findings contribute to this ongoing debate by identifying specific thresholds where the risk of mortality changes significantly, highlighting the need for further research to clarify the optimal range of serum 25(OH)D for various populations. Additionally, our stratified analyses revealed that the race subgroup showed *p*-values close to the significance threshold for both all-cause and CVD mortality. This suggests that individuals from certain race backgrounds may benefit from further investigation. This finding aligns with previous studies that have noted ethnic disparities in vitamin D metabolism and health outcomes, indicating that genetic, environmental, and lifestyle factors may influence the relationship between vitamin D and mortality (32). For instance, Artaza et al. reported that vitamin D deficiency was particularly prevalent among African American populations, which is consistent with their elevated incidence of CVD (33). Furthermore, the relationship between vitamin D levels and mortality has been investigated across various populations. The D-Health Trial found no significant association between vitamin D levels and all-cause mortality in a cohort of Australian individuals (34). Similarly, the VITAL trial did not demonstrate a significant protective effect of vitamin D supplementation against cancer and CVD among US adults (8). These discrepancies highlight the complexity of vitamin D’s role in health and the need for further investigation into the underlying mechanisms.

Understanding the mechanisms by which 25(OH)D influences mortality risk is crucial for accurately interpreting our findings. While vitamin D is primarily recognized for its role in calcium homeostasis and bone remodeling, its effects extend far beyond skeletal health. A fundamental mechanism associated with sarcopenia is the impact of vitamin D on muscle function. Vitamin D receptors are abundantly present in muscle tissue, and considerable evidence indicates that sufficient levels of vitamin D are correlated with enhanced muscle strength and function (4, 35). Specifically, vitamin D facilitates muscle protein synthesis and may promote muscle regeneration, both of which are especially vital for older adults at an increased risk of sarcopenia (36, 37). In parallel, lifestyle interventions such as regular physical activity, specifically programs combining resistance training and aerobic exercise, demonstrate independent efficacy in sarcopenia prevention (38). These complementary biological and behavioral strategies collectively address the multifactorial pathogenesis of muscle loss, which significantly elevates risks of morbidity and mortality in aging populations. The loss of muscle mass and physical strength typical of sarcopenia significantly raises the risk of morbidity and mortality, emphasizing the vital importance of vitamin D for this population (4, 39). Another important mechanism involves the anti-inflammatory function of vitamin D, as chronic inflammation is a widely recognized risk factor for various diseases, including CVD, diabetes, and other age-related conditions. Vitamin D modulates the immune response and has been shown to lower the concentrations of pro-inflammatory cytokines, thereby fostering a more favorable inflammatory profile (40, 41). This anti-inflammatory action may mitigate the risks of chronic diseases prevalent among individuals with insufficient vitamin D levels, consequently reducing mortality risk. Emerging evidence suggests that vitamin D also exerts direct effects on cardiovascular health through its actions on the vascular system (42, 43). Vitamin D deficiency has been linked to adverse cardiovascular events, potentially via pathways involving endothelial function, blood pressure regulation, and modulation of vascular inflammation (44). Furthermore, vitamin D is indispensable for the preservation of bone health, as insufficiency can induce osteoporosis and elevate the incidence of fractures, particularly among the geriatric population (45). Falls and resultant fractures are significant contributors to morbidity and mortality within this demographic group (46). By maintaining adequate concentration of vitamin D, it is possible to enhance bone density, thereby reducing the likelihood of falls and indirectly impacting mortality outcomes. This highlights the multifactorial role of vitamin D in optimizing health across various biological systems.

Our research has certain strengths and constraints. The observational nature of the NHANES data precludes definitive conclusions regarding causality. Although adjustments were made for numerous confounding variables, the possibility of residual confounding cannot be entirely eliminated. Besides, the reliance on self-reported data concerning chronic disease histories may introduce biases that affect the findings. Furthermore, the analysis was based on data from the NHANES 2001–2006 cohort, while the biological mechanism are likely stable over time, secular trends in lifestyle factors could theoretically alter this association. Future research employing longitudinal designs and objective assessments of 25(OH)D concentration is necessary to validate these results and clarify the underlying causal pathways. Investigations should prioritize elucidating the pathway through which of 25(OH)D affects mortality risk. Longitudinal studies examining the impact of vitamin D supplementation on clinical outcomes, particularly within sarcopenia populations at heightened risk for deficiency, will be vital. Additionally, assessing the interactions between 25(OH)D and other lifestyle factors, such as physical activity and dietary patterns, may yield further insights into the intricate relationships among 25(OH)D, general well-being, and mortality.

## 5 Conclusion

In conclusion, our study emphasizes the significant association between serum 25(OH)D concentration and mortality outcomes in a cohort of sarcopenia participants from NHANES. The findings underscore the importance of maintaining adequate 25(OH)D concentration for overall health and longevity, particularly in the context of sarcopenia. As the evidence continues to accumulate, it is imperative that public health strategies prioritize the prevention and management of vitamin D deficiency to improve health outcomes across populations.

## Data Availability

The original contributions presented in the study are included in the article/Supplementary material, further inquiries can be directed to the corresponding author.
