# Supplementary material for: Association of 25-hydroxyvitamin D with all-cause and cardiovascular mortality among individuals with sarcopenia: evidence from the NHANES 2001–2006
Source: Front Nutr. 2025 May 16;12:1562897. doi: 10.3389/fnut.2025.1562897 (PMC12124123; doi:10.3389/fnut.2025.1562897)
Supplement: Supplementary file 1 [file Table_1.docx]

Supplementary TABLE S1. Multivariable Cox regression analysis for Mortality after Excluding Participants with History of CHD

|  | **Serum 25(OH)D concentration (nmol/L)** | | | | | Per one-unit increment in  ln-transformed 25(OH)D |
| --- | --- | --- | --- | --- | --- | --- |
|  | <25.00 | 25.00-49.99 | 50.00-74.99 | ≥75.00 | P for trend |  |
| All-cause mortality |  |  |  |  |  |  |
| Model 1 | Reference | 0.45 (0.31, 0.67) <0.0001 | 0.34 (0.23, 0.51) <0.0001 | 0.39 (0.26, 0.60) <0.0001 | <0.0001 | 0.56 (0.45, 0.71) <0.0001 |
| Model 2 | Reference | 0.49 (0.33, 0.72) 0.0003 | 0.36 (0.24, 0.53) <0.0001 | 0.44 (0.28, 0.67) 0.0002 | 0.0005 | 0.57 (0.45, 0.73) <0.0001 |
| Model 3 | Reference | 0.55 (0.38, 0.81) 0.0026 | 0.42 (0.29, 0.63) <0.0001 | 0.49 (0.32, 0.76) 0.0014 | 0.0015 | 0.61 (0.49, 0.78) <0.0001 |
| CVD  mortality |  |  |  |  |  |  |
| Model 1 | Reference | 0.25 (0.13, 0.50) 0.0001 | 0.18 (0.09, 0.37) <0.0001 | 0.23 (0.10, 0.50) 0.0002 | 0.0683 | 0.53 (0.33, 0.84) 0.0073 |
| Model 2 | Reference | 0.27 (0.13, 0.54) 0.0003 | 0.19 (0.09, 0.40) <0.0001 | 0.25 (0.11, 0.57) 0.0008 | 0.1901 | 0.58 (0.36, 0.95) 0.0301 |
| Model 3 | Reference | 0.33 (0.16, 0.68) 0.0026 | 0.26 (0.13, 0.55) 0.0004 | 0.33 (0.15, 0.74) 0.0076 | 0.3304 | 0.66 (0.41, 1.07) 0.0949 |

Model 1: adjust for gender, age, race, education, PIR. Model 2: adjust for gender, age, race, education, PIR, BMI, smoking history, drinking status, hypertension, diabetes, stroke, bronchitis and cancer. Model 3: adjust for gender, age, race, education, PIR, BMI, smoking history, drinking status, hypertension, diabetes, stroke, bronchitis, cancer, serum albumin, ALT, AST, eGFR, glycohemoglobin, hemoglobin, TC and HDL-C. Abbreviation: 95% CI, 95% confidence interval; OR, odds ratio; 25(OH)D, 25-hydroxyvitamin D; CVD, cardiovascular disease. *p*<0.05 was considered statistically significant.

Supplementary TABLE S2. Multivariable Cox regression analysis for Mortality after Excluding Participants with History of stroke

|  | **Serum 25(OH)D concentration (nmol/L)** | | | | | Per one-unit increment in  ln-transformed 25(OH)D |
| --- | --- | --- | --- | --- | --- | --- |
|  | <25.00 | 25.00-49.99 | 50.00-74.99 | ≥75.00 | P for trend |  |
| All-cause mortality |  |  |  |  |  |  |
| Model 1 | Reference | 0.55 (0.37, 0.81) 0.0026 | 0.42 (0.28, 0.63) <0.0001 | 0.46 (0.30, 0.71) 0.0004 | <0.0001 | 0.57 (0.45, 0.71) <0.0001 |
| Model 2 | Reference | 0.61 (0.41, 0.90) 0.0137 | 0.47 (0.31, 0.70) 0.0003 | 0.54 (0.35, 0.84) 0.0058 | 0.0012 | 0.60 (0.48, 0.76) <0.0001 |
| Model 3 | Reference | 0.67 (0.45, 1.00) 0.0489 | 0.53 (0.35, 0.78) 0.0016 | 0.59 (0.38, 0.92) 0.0197 | 0.0017 | 0.63 (0.50, 0.79) <0.0001 |
| CVD  mortality |  |  |  |  |  |  |
| Model 1 | Reference | 0.29 (0.14, 0.59) 0.0006 | 0.22 (0.11, 0.45) <0.0001 | 0.23 (0.10, 0.50) 0.0002 | 0.0201 | 0.49 (0.31, 0.77) 0.0022 |
| Model 2 | Reference | 0.33 (0.16, 0.67) 0.0022 | 0.26 (0.12, 0.53) 0.0002 | 0.29 (0.13, 0.64) 0.0023 | 0.1413 | 0.59 (0.37, 0.94) 0.0277 |
| Model 3 | Reference | 0.39 (0.19, 0.80) 0.0097 | 0.32 (0.16, 0.66) 0.0021 | 0.36 (0.16, 0.81) 0.0131 | 0.2461 | 0.65 (0.41, 1.04) 0.0733 |

Model 1: adjust for gender, age, race, education, PIR. Model 2: adjust for gender, age, race, education, PIR, BMI, smoking history, drinking status, hypertension, CHD, diabetes, bronchitis and cancer. Model 3: adjust for gender, age, race, education, PIR, BMI, smoking history, drinking status, hypertension, CHD, diabetes, bronchitis, cancer, serum albumin, ALT, AST, eGFR, glycohemoglobin, hemoglobin, TC and HDL-C. Abbreviation: 95% CI, 95% confidence interval; OR, odds ratio; 25(OH)D, 25-hydroxyvitamin D; CVD, cardiovascular disease. *p*<0.05 was considered statistically significant.

Supplementary TABLE S3. Multivariable Cox regression analysis for Mortality after Excluding Participants with History of bronchitis

|  | **Serum 25(OH)D concentration (nmol/L)** | | | | | Per one-unit increment in  ln-transformed 25(OH)D |
| --- | --- | --- | --- | --- | --- | --- |
|  | <25.00 | 25.00-49.99 | 50.00-74.99 | ≥75.00 | P for trend |  |
| All-cause mortality |  |  |  |  |  |  |
| Model 1 | Reference | 0.41 (0.28, 0.59) <0.0001 | 0.32 (0.22, 0.47) <0.0001 | 0.36 (0.24, 0.54) <0.0001 | <0.0001 | 0.55 (0.44, 0.69) <0.0001 |
| Model 2 | Reference | 0.47 (0.33, 0.68) <0.0001 | 0.38 (0.26, 0.55) <0.0001 | 0.44 (0.29, 0.66) <0.0001 | 0.0010 | 0.60 (0.48, 0.75) <0.0001 |
| Model 3 | Reference | 0.54 (0.37, 0.78) 0.0010 | 0.44 (0.30, 0.64) <0.0001 | 0.49 (0.32, 0.73) 0.0005 | 0.0011 | 0.62 (0.50, 0.78) <0.0001 |
| CVD  mortality |  |  |  |  |  |  |
| Model 1 | Reference | 0.20 (0.11, 0.39) <0.0001 | 0.16 (0.08, 0.31) <0.0001 | 0.18 (0.09, 0.36) <0.0001 | 0.0181 | 0.48 (0.31, 0.75) 0.0013 |
| Model 2 | Reference | 0.25 (0.13, 0.49) <0.0001 | 0.21 (0.11, 0.40) <0.0001 | 0.24 (0.12, 0.50) 0.0001 | 0.1018 | 0.56 (0.35, 0.89) 0.0137 |
| Model 3 | Reference | 0.31 (0.16, 0.60) 0.0005 | 0.27 (0.14, 0.52) <0.0001 | 0.29 (0.14, 0.61) 0.0010 | 0.1408 | 0.61 (0.39, 0.96) 0.0314 |

Model 1: adjust for gender, age, race, education, PIR. Model 2: adjust for gender, age, race, education, PIR, BMI, smoking history, drinking status, hypertension, CHD, diabetes, stroke and cancer. Model 3: adjust for gender, age, race, education, PIR, BMI, smoking history, drinking status, hypertension, CHD, diabetes, stroke, cancer, serum albumin, ALT, AST, eGFR, glycohemoglobin, hemoglobin, TC and HDL-C. Abbreviation: 95% CI, 95% confidence interval; OR, odds ratio; 25(OH)D, 25-hydroxyvitamin D; CVD, cardiovascular disease. *p*<0.05 was considered statistically significant.

Supplementary TABLE S4. Multivariable Cox regression analysis for Mortality after Excluding Participants with History of cancer

|  | **Serum 25(OH)D concentration (nmol/L)** | | | | | Per one-unit increment in  ln-transformed 25(OH)D |
| --- | --- | --- | --- | --- | --- | --- |
|  | <25.00 | 25.00-49.99 | 50.00-74.99 | ≥75.00 | P for trend |  |
| All-cause mortality |  |  |  |  |  |  |
| Model 1 | Reference | 0.47 (0.33, 0.69) <0.0001 | 0.36 (0.25, 0.52) <0.0001 | 0.41 (0.27, 0.61) <0.0001 | <0.0001 | 0.53 (0.42, 0.67) <0.0001 |
| Model 2 | Reference | 0.51 (0.35, 0.75) 0.0005 | 0.38 (0.26, 0.56) <0.0001 | 0.46 (0.31, 0.71) 0.0003 | 0.0003 | 0.55 (0.43, 0.70) <0.0001 |
| Model 3 | Reference | 0.57 (0.39, 0.83) 0.0030 | 0.43 (0.29, 0.63) <0.0001 | 0.49 (0.32, 0.75) 0.0010 | 0.0003 | 0.57 (0.45, 0.72) <0.0001 |
| CVD  mortality |  |  |  |  |  |  |
| Model 1 | Reference | 0.27 (0.14, 0.52) <0.0001 | 0.20 (0.11, 0.40) <0.0001 | 0.23 (0.11, 0.48) <0.0001 | 0.0495 | 0.52 (0.33, 0.83) 0.0059 |
| Model 2 | Reference | 0.31 (0.16, 0.60) 0.0005 | 0.23 (0.12, 0.45) <0.0001 | 0.28 (0.13, 0.60) 0.0009 | 0.1442 | 0.58 (0.36, 0.93) 0.0240 |
| Model 3 | Reference | 0.36 (0.19, 0.71) 0.0031 | 0.29 (0.15, 0.56) 0.0002 | 0.34 (0.16, 0.72) 0.0050 | 0.2747 | 0.65 (0.40, 1.03) 0.0683 |

Model 1: adjust for gender, age, race, education, PIR. Model 2: adjust for gender, age, race, education, PIR, BMI, smoking history, drinking status, hypertension, CHD, diabetes, stroke and bronchitis. Model 3: adjust for gender, age, race, education, PIR, BMI, smoking history, drinking status, hypertension, CHD, diabetes, stroke, bronchitis, serum albumin, ALT, AST, eGFR, glycohemoglobin, hemoglobin, TC and HDL-C. Abbreviation: 95% CI, 95% confidence interval; OR, odds ratio; 25(OH)D, 25-hydroxyvitamin D; CVD, cardiovascular disease. *p*<0.05 was considered statistically significant.

Supplementary TABLE S5. Multivariable Cox regression analysis for Mortality after Excluding Participants with less than 2-year of follow-up

|  | **Serum 25(OH)D concentration (nmol/L)** | | | | | Per one-unit increment in  ln-transformed 25(OH)D |
| --- | --- | --- | --- | --- | --- | --- |
|  | <25.00 | 25.00-49.99 | 50.00-74.99 | ≥75.00 | P for trend |  |
| All-cause mortality |  |  |  |  |  |  |
| Model 1 | Reference | 0.50 (0.34, 0.73) 0.0003 | 0.39 (0.27, 0.58) <0.0001 | 0.45 (0.29, 0.68) 0.0002 | 0.0009 | 0.61 (0.48, 0.76) <0.0001 |
| Model 2 | Reference | 0.56 (0.38, 0.83) 0.0033 | 0.44 (0.30, 0.65) <0.0001 | 0.52 (0.34, 0.80) 0.0027 | 0.0046 | 0.63 (0.50, 0.80) 0.0001 |
| Model 3 | Reference | 0.61 (0.42, 0.90) 0.0125 | 0.48 (0.33, 0.71) 0.0003 | 0.55 (0.36, 0.84) 0.0058 | 0.0039 | 0.65 (0.52, 0.82) 0.0002 |
| CVD  mortality |  |  |  |  |  |  |
| Model 1 | Reference | 0.26 (0.13, 0.52) 0.0001 | 0.18 (0.09, 0.36) <0.0001 | 0.22 (0.10, 0.47) <0.0001 | 0.0438 | 0.53 (0.34, 0.85) 0.0075 |
| Model 2 | Reference | 0.30 (0.15, 0.60) 0.0006 | 0.20 (0.10, 0.41) <0.0001 | 0.25 (0.12, 0.54) 0.0005 | 0.0861 | 0.57 (0.35, 0.90) 0.0174 |
| Model 3 | Reference | 0.36 (0.18, 0.72) 0.0036 | 0.25 (0.12, 0.50) <0.0001 | 0.30 (0.14, 0.65) 0.0022 | 0.1112 | 0.61 (0.38, 0.96) 0.0335 |

Model 1: adjust for gender, age, race, education, PIR. Model 2: adjust for gender, age, race, education, PIR, BMI, smoking history, drinking status, hypertension, CHD, diabetes, stroke, bronchitis and cancer. Model 3: adjust for gender, age, race, education, PIR, BMI, smoking history, drinking status, hypertension, CHD, diabetes, stroke, bronchitis, cancer, serum albumin, ALT, AST, eGFR, glycohemoglobin, hemoglobin, TC and HDL-C. Abbreviation: 95% CI, 95% confidence interval; OR, odds ratio; 25(OH)D, 25-hydroxyvitamin D; CVD, cardiovascular disease. *p*<0.05 was considered statistically significant.

Supplementary TABLE S6. Multivariable Cox regression analysis for Mortality after Excluding Participants with extreme values of the 25(OH)D

|  | **Serum 25(OH)D concentration (nmol/L)** | | | | | Per one-unit increment in  ln-transformed 25(OH)D |
| --- | --- | --- | --- | --- | --- | --- |
|  | <25.00 | 25.00-49.99 | 50.00-74.99 | ≥75.00 | P for trend |  |
| All-cause mortality |  |  |  |  |  |  |
| Model 1 | Reference | 0.46 (0.33, 0.65) <0.0001 | 0.36 (0.25, 0.51) <0.0001 | 0.42 (0.28, 0.62) <0.0001 | <0.0001 | 0.57 (0.46, 0.71) <0.0001 |
| Model 2 | Reference | 0.52 (0.37, 0.75) 0.0003 | 0.41 (0.28, 0.58) <0.0001 | 0.49 (0.33, 0.72) 0.0003 | 0.0008 | 0.60 (0.48, 0.75) <0.0001 |
| Model 3 | Reference | 0.59 (0.41, 0.83) 0.0028 | 0.46 (0.32, 0.66) <0.0001 | 0.54 (0.36, 0.79) 0.0018 | 0.0019 | 0.64 (0.51, 0.79) <0.0001 |
| CVD  mortality |  |  |  |  |  |  |
| Model 1 | Reference | 0.24 (0.13, 0.44) <0.0001 | 0.18 (0.10, 0.34) <0.0001 | 0.21 (0.11, 0.42) <0.0001 | 0.0190 | 0.50 (0.32, 0.76) 0.0011 |
| Model 2 | Reference | 0.28 (0.15, 0.52) <0.0001 | 0.22 (0.12, 0.40) <0.0001 | 0.26 (0.13, 0.51) 0.0001 | 0.0815 | 0.56 (0.36, 0.86) 0.0087 |
| Model 3 | Reference | 0.34 (0.18, 0.63) 0.0006 | 0.27 (0.15, 0.51) <0.0001 | 0.32 (0.16, 0.65) 0.0014 | 0.1812 | 0.63 (0.41, 0.96) 0.0332 |

Model 1: adjust for gender, age, race, education, PIR. Model 2: adjust for gender, age, race, education, PIR, BMI, smoking history, drinking status, hypertension, CHD, diabetes, stroke, bronchitis and cancer. Model 3: adjust for gender, age, race, education, PIR, BMI, smoking history, drinking status, hypertension, CHD, diabetes, stroke, bronchitis, cancer, serum albumin, ALT, AST, eGFR, glycohemoglobin, hemoglobin, TC and HDL-C. Abbreviation: 95% CI, 95% confidence interval; OR, odds ratio; 25(OH)D, 25-hydroxyvitamin D; CVD, cardiovascular disease. *p*<0.05 was considered statistically significant.

Supplementary TABLE S7. Missing of variables

| Variables | Missing (n) | Missing proportion (%) |
| --- | --- | --- |
| Hb | 1 | 0.06 |
| diabetes | 3 | 0.18 |
| TC | 3 | 0.18 |
| HDL | 4 | 0.24 |
| Glycohemoglobin | 5 | 0.30 |
| albumin | 11 | 0.66 |
| eGFR | 11 | 0.66 |
| ALT | 16 | 0.96 |
| AST | 16 | 0.96 |
| hypertension | 27 | 1.62 |
| cancer | 47 | 2.82 |
| education | 48 | 2.88 |
| smoke | 49 | 2.94 |
| stroke | 49 | 2.94 |
| bronchitis | 55 | 3.30 |
| CHD | 71 | 4.26 |
| PIR | 92 | 5.52 |
| Drink | 139 | 8.34 |


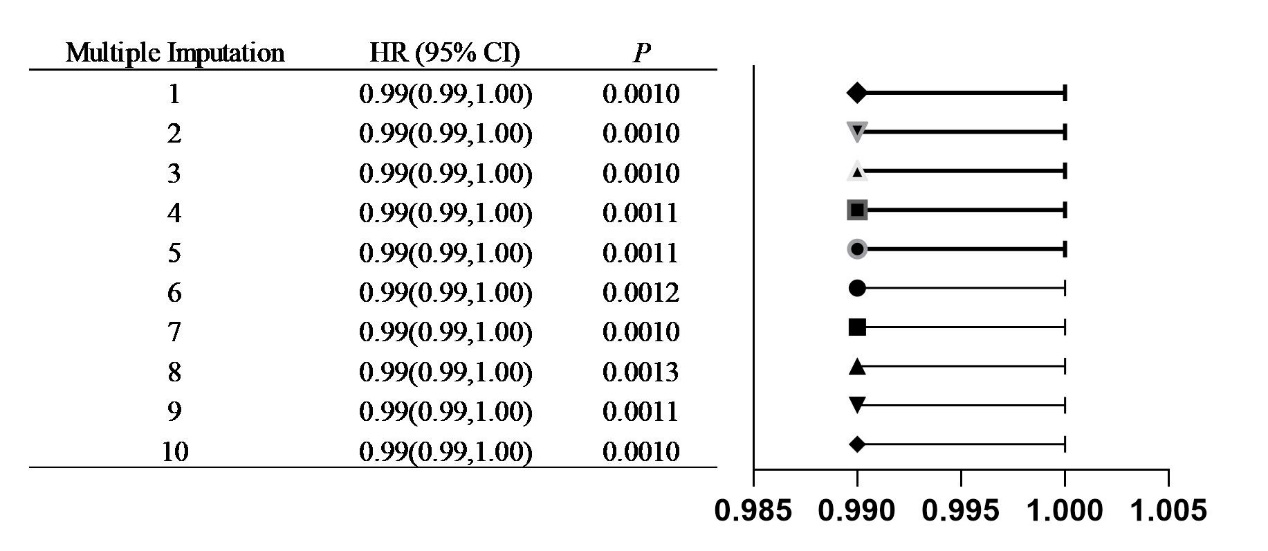


Supplenmentary Figure 1. Results of HR for all-cause mortality after multiple imputations (10 times) of missing data

**
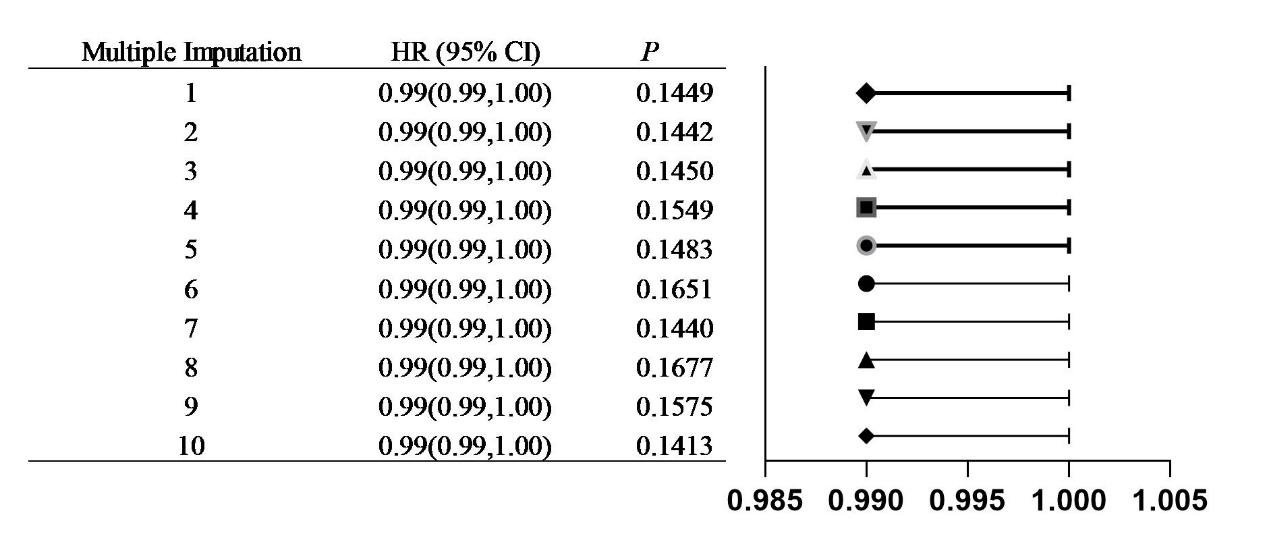
**

Supplenmentary Figure 2. Results of HR for CVD mortality after multiple imputations (10 times) of missing data
